# Supplementary material for: Detection of Circulating VZV-Glycoprotein E-Specific Antibodies by Chemiluminescent Immunoassay (CLIA) for Varicella–Zoster Diagnosis
Source: Pathogens. 2022 Jan 5;11(1):66. doi: 10.3390/pathogens11010066 (PMC8778750; doi:10.3390/pathogens11010066)
Supplement: Supplementary file 1 [file pathogens-11-00066-s001.zip › pathogens-1437564-supplementary.pdf]

Supplementary data

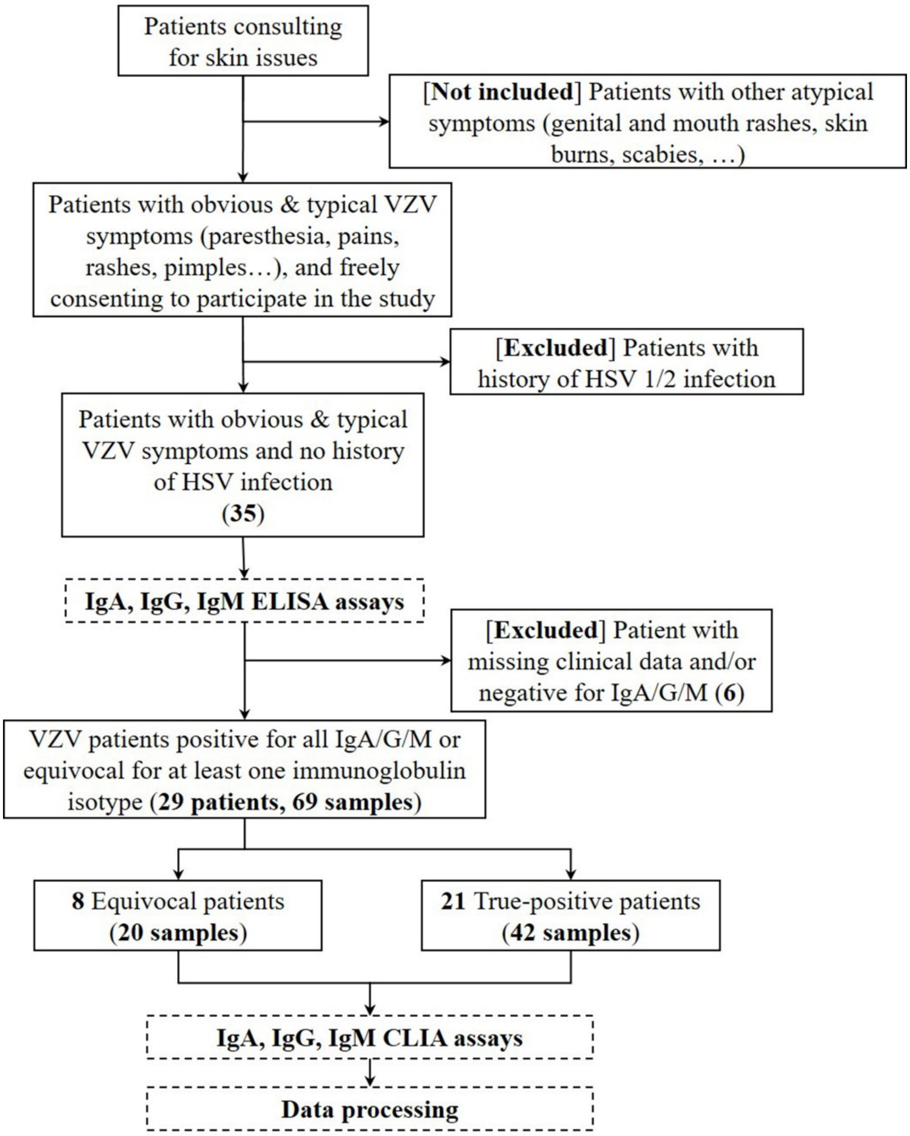

Supplementary Figure S1: Flow chart of patient inclusion and analyses. None of the retained VZV-patients had been vaccinated with VZV vaccine.

Supplementary Table S1: Epidemiological and clinical patient data

N°	Sex	Age	Symp. onset	Symptoms	Organ transplant	Other infectious diseases
**1**	M	72	7	Fever and rashes, and discomfort for more than 7 days	no	–
**2**	F	37	4	4 days of lesions on upper left abdomen. 1 day of pain	no	no
**3**	M	79	6	Lesions on left upper limb and front chest wall for 6 days with pain	no	no
**4**	M	29	–		no	no
**5**	M	38	7	Head and neck pain for 7 days and lesions for 4 days	no	no
**6**	M	33	2	Redness and swelling of right eye with pain for 2 days	no	no
**7**	F	48	3	Headache for 3 days with redness of right eye frame for 1 day	no	no
**8**	M	35	5	Lesions on right lumbodorsal region with pain for 5 days	no	no
**9**	M	31	6	Redness and swelling around left frame for 6 days with aggravation for 2 days	no	no
**10**	F	63	6	Left parietal temporal rash with pain for 6 days	no	no
**11**	M	69	4	Left head and neck rash for 4 days	no	no
**12**	M	–	8	Left head and neck rash for 8 days	no	no
**13**	F	20	3	Rash on right eyelid with pain for 3 days	no	Hepatitis B
**14**	M	63	3	Right facial rash with pain for 3 days	no	no
**15**	M	26	2	Rash on left shoulder with pain for 2 days	no	no
**16**	F	65	15	Rash on right lower back with pain for 15 days	no	no
**17**	F	27	7	Fever for 1 week. lesions on left chest and back with pain for 5 days	no	no
**18**	M	79	14	Rash on right side of head with pain for 2 weeks	no	no
**19**	M	70	7	Rash on left shoulder, back, left upper limb for 1 week and pain for 3 days	no	no
**20**	M	73	2	Rash on left side of waist and abdomen for 2 days	no	Hepatitis B
**21**	M	38	15	Rash on right side of waist with pains	no	no
**22**	M	63	5	Chest and back rash with fever for 5 days	no	no
**23**	F	28	5	Right chest and back rash with pain for 5 days	no	no
**24**	M	48	2	Rash on left side back for 2 days	no	Hepatitis A, Hepatitis E
**25**	M	82	9	Neck rash 9 days	no	no
**26**	F	56	10	Left head pain for 10 days. left head and face rash for 8 days	no	no
**27**	F	70	5	Pain in left chest and back for 1 week and rash for 5 days	no	no
**28**	M	38	15	Fever and localized rashes	no	no
**29**	F	45	7	Rashes on left side of waist, abdomen and back for 5 days, fever for 7 days	no	no

The sample from these patients were collecting at the visiting time, for this study. No patient had received VZV vaccine before the consulting time. Moreover, no skin diseases has been reported before by any of the patients.

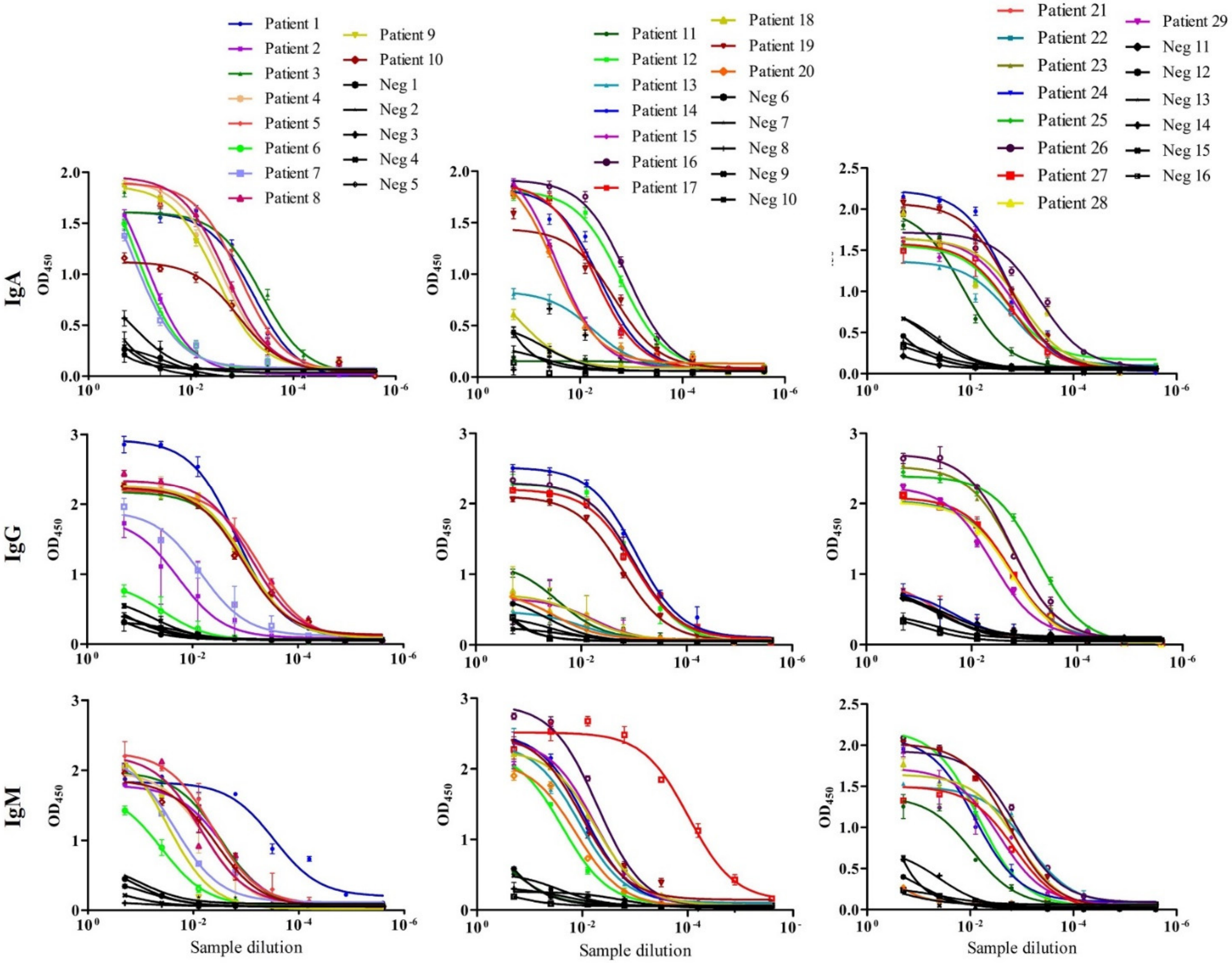

Supplementary Figure S2. ELISA results of all the 29 included patients. All the patients sample were performed thrice, with 5-fold serial dilution from for each sample. All the negative controls are shown in black curves, while the patients are shown in colored curves.

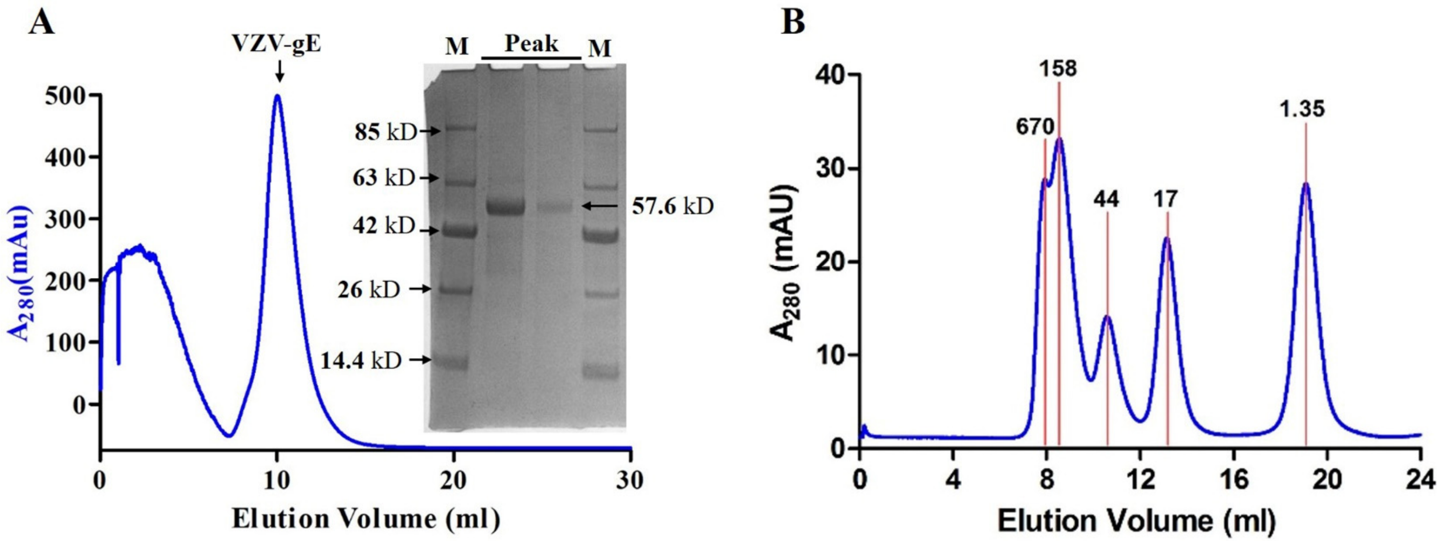

Supplementary Figure S3. Purification of VZV-gE recombinant protein using baculovirus vector expression system (BVES). **A.** The recombinant protein was obtained from supernatant of Hi5 cell line culture, and purified. The pure protein was characterized through 10% SDS-PAGE based molecular weight (kDa) separation and determined using protein market (M). After staining with Coomassie blue dye, the gel was carefully washed and the purified band (57 kDa) was visualized on fluorescent light. **B.** A standard elution curve obtained from known molecular weight protein using the same size-exclusion chromatography (SEC) device was used to characterize the monomeric state of the purified VZV-gE protein.

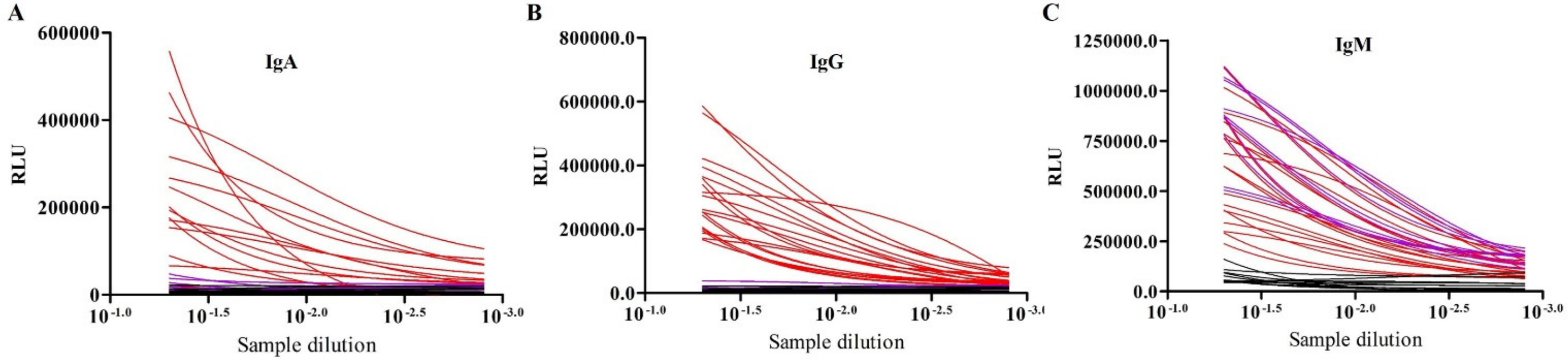

Supplementary Figure S4. Validation of VZV-gE specific IgA (**A**), IgG (**B**), and IgM (**C**) detection based on CLIA approach. 29 included patient samples were randomly selected and 2-fold serially diluted (from 1/20) to assess the ability of the purified gE protein to detect IgG and IgM antibodies using automated CLIA. The relative light unit (RLU) obtained for all the patients were transform and fit using GraphPad Prism 5. The negative controls are shown in black curves, the ELISA based equivocal are colored in pink-purple, and the positive are colored in red. RLU: relative light unit. This validated the diagnostic approach.
